# Between Pleasure and Contentment: Evolutionary Dynamics of Some Possible Parameters of Happiness

**DOI:** 10.1371/journal.pone.0153193

**Published:** 2016-05-04

**Authors:** Yue Gao, Shimon Edelman

**Affiliations:** 1 Dept. of Computer Science, Cornell University, Ithaca, NY, United States of America; 2 Dept. of Psychology, Cornell University, Ithaca, NY, United States of America; Beihang University, CHINA

## Abstract

We offer and test a simple operationalization of hedonic and eudaimonic well-being (“happiness”) as mediating variables that link outcomes to motivation. In six evolutionary agent-based simulation experiments, we compared the relative performance of agents endowed with different combinations of happiness-related traits (parameter values), under four types of environmental conditions. We found (i) that the effects of attaching more weight to longer-term than to momentary happiness and of extending the memory for past happiness are both stronger in an environment where food is scarce; (ii) that in such an environment “relative consumption,” in which the agent’s well-being is negatively affected by that of its neighbors, is more detrimental to survival when food is scarce; and (iii) that having a positive outlook, under which agents’ longer-term happiness is increased by positive events more than it is decreased by negative ones, is generally advantageous.

## Introduction

Happiness and other emotional states play a central role in human existence by mediating the regulation of behavior [1–4]. While this construal of emotions was originally motivated by classical control theory [5], it fits well within the emerging integrative computational framework for understanding the brain/mind, which holds that minds are bundles of computational processes implemented by embodied and physically and socially situated brains [6].

The computational framework allows one to put forward and test very explicit functional models of emotions. In this paper, we use such a model to investigate, in an evolutionary setting, a series of questions pertaining to happiness. These include (i) the role of balancing momentary well-being against longer-term contentment (the “happiness of pursuit” [7]); (ii) the effects of drawing a contrast between oneself and one’s social circle (what economists term “relative consumption” [8, 9]; cf. the concept of social comparison [10, 11]); and (iii) the adaptive role of differential sensitivity to positive and negative turns in momentary well-being.

Happiness and other emotions are experienced subjectively; indeed, it is the subjective well-being (SWB) that psychologists who study happiness require that the participants in their experiments report [12]. To study happiness in simple computational models that are obviously devoid of any phenomenality or subjectivity [13, 14], we need an objective “handle” onto emotions, which would make explicit their role in behavior and evolution. For this purpose, it suffices to limit our consideration to the *valuation* aspects of emotions [12, 15]. Our theoretical approach is therefore based on the following set of interlinked premises:

1. Subjectivity, including *phenomenal awareness*, evolves to serve as an effective tool for connecting *action outcomes* to *motivation* [16, 17].
2. Subjective *valuation* and the affective states or *emotions* that mediate it, including *happiness*, serve as a key pressure point through which *evolution* acts on the mind [7, 18, 19].
3. Emotions are information channels whereby phenomenal states and valuation processes motivate decisions, *regulate behavior* [15, 20], and fine-tune learning [21]. As such, emotions pervade all of cognition [3, 20, 22, 23].
4. There exist *heritable individual differences* in the contributions of cognitive, motivational, and affective states and processes to well-being [24, 25].
5. Happiness-related traits affect the evolutionary *fitness* of their carriers [26], in ways that depend on physical and social circumstances.

In the remainder of this paper, we report the results of six experiments in which we explored the evolutionary dynamics of happiness as a mediator between action outcomes and life evaluation on the one hand and action selection on the other hand. We begin with a brief review of related work and of the literature that supports our working assumptions.

## Related work and the present approach

In this section, we briefly discuss (i) the role of emotions and motivation in driving behavior; (ii) agent-based evolutionary simulation as a tool for studying these topics; (iii) the hedonic and eudaimonic components of happiness and their further factorization; (iv) social context as a key factor; (v) the role of variables that control the temporal dynamics of hedonic and eudaimonic well-being.

### Emotion, motivation, action

Behavior is considered to be motivated if it is at least partly determined by its expected consequences [2]. Because the consequences of a planned or future action are not available prior to its execution, the control of motivated behavior involves internal states, which represent goals or expected outcomes. For evolutionary reasons briefly mentioned above, some such states come to be experienced as emotional. Specifically, emotional states, including happiness, convey valuational information, about which the agent by definition cares, and which therefore serves as an effective motivational mechanism for action selection [2, 27]. Motivation is influenced by many types of emotions in addition to happiness. Our goal in the present paper is not to compare the effects of different emotions or to model them; rather, we are interested specifically in the effects of hedonic and eudaimonic well-being.

### Agent-based evolutionary simulation modeling

While in principle it is possible to study the complex interplay of emotions, motivation, and evolution analytically (e.g., [28]), it is often more practical to do so by resorting to simulation, using an evolutionary agent-based modeling (ABM) approach [29–31]. In ABM, simulated actors (agents) carrying various traits of interest share an environment in which they undertake actions and compete for resources; the agent’s cumulative outcomes determine its fitness, which in turn affects its chances for reproduction. The effectiveness of traits can then be assessed by tracking their prevalence in the population over evolutionary time.

The ABM approach has been previously applied to the study of emotions and motivation (e.g., [32–34]). For instance, Malfaz and Salichs [32] used it to model motivation as a combination of internal drives and external stimuli. The drive variables were energy, thirst, health, sociability, and fear; the external stimuli were water, food, and the presence of other agents.

In an ABM setting, the relative contributions of the various factors that jointly affect behavior can be tuned using any of a number of learning approaches, in particular reinforcement learning (RL) [35–37], as it was done in [32]. Because our goal in the present work was to determine the effects of specific combinations of the values of relevant parameters, we chose not to allow our agents to learn; a companion paper (Gao and Edelman, in preparation) will report results from a learning-enabled version of our model.

### The factorization of happiness

A distinction is commonly drawn between two major components of happiness, operationalized as subjective well-being: the *hedonic* component, estimated through responses to questions such as “How happy are you right now?”, and the *eudaimonic* component, based on responses to questions such as “How happy are you with your life in general?” [12, 38]. To be useful in an ABM setting, each of these components must be given an explicit mathematical definition in terms of the independent variables of the model.

The precise form of such a definition can itself be the subject of an investigation. For instance, Rutledge et al. [39] considered various ways of quantifying subjects’ momentary hedonic SWB *H* in response to outcomes in an economic game and showed that it is best modeled by combining current task earnings (*CR*), recent reward expectations (*EV*), and reward prediction errors (*RPE*), as follows:

$$\begin{array}{c} H=w_{0}+w_{1}\sum_{t=1}^{t}\gamma^{t-j}CR_{j}+w_{2}\sum_{j=1}^{t}\gamma^{t-j}EV_{j}+w_{3}\sum_{j=1}^{t}\gamma^{t-j}RPE_{j} \end{array}$$ (1)

where *t* is the number of days in memory and 0 ≤ *γ* ≤ 1 is a forgetting factor that makes days in more recent trials more influential than those in earlier trials. In the present project, we likewise assume that happiness is related to a time average of outcomes (excluding the RL factors, as noted above) and explore the evolutionary dynamics of traits that control the contributions of momentary and time-averaged values.

### The social dimension of hedonic well-being

In behavioral economics, it is well known that people’s perceived conditions with regard to the so-called positional goods depend on those of their social circle or comparison group [8, 9]. Intuitively, subjects can be more or less happy with the same absolute level of a positional good (say, a house of a given size), depending on the levels of their neighbors. The study of Baggio and Papyrakis [33] focused on the effects of this type of social comparison. Specifically, they assumed that the hedonic SWB *H* of agent *k* in a given year *t* depended positively on their own income that year, $Y_{k}^{t}$, and negatively on the average social income for the same period, $\bar{Y^{t}}$, as well as its own income in the previous year, $Y_{k}^{t-1}$:

$$\begin{array}{c} \begin{array}{c} H_{k}^{t}=Y_{k}^{t}-\beta Y_{k}^{t-1}-\alpha \bar{Y^{t}} \end{array} \end{array}$$ (2)

where *α* and *β* are sensitivity parameters in the range of [0, 1]. They then examined the dependence of *H* on individual income and social comparison (economic inequality) by considering the effects of the sensitivity parameters in an ABM setting.

### The dynamic relationships between hedonic and eudaimonic well-being

Intuitively, the eudaimonic SWB, which we shall refer to as *E*, is expected to be related to the integral of the hedonic SWB, *H*, but the details of this relationship are up to the modeler. Taking inspiration from Strogatz’s model of love [40], Sprott [41, 42] offered the following second-order linear differential equation for cumulative happiness, which he denoted by *R*:

$$\begin{array}{c} \begin{array}{c} \frac{d^{2}R}{dt^{2}}+\beta \frac{dR}{dt}+R=F\left(t\right) \end{array} \end{array}$$ (3)

where *F*(*t*) is a time-dependent function that quantifies the effects of external events. This equation can be re-written in terms of the momentary well-being, which we call *H*, in the following form: $\frac{dH}{dt}+\beta H+\int H=F(t)$. Note that this formulation makes explicit the dependence of happiness on outcomes, as well as on its history and changes (the integral and derivative terms, respectively).

## Methods

We now turn to the description of our own work, which uses evolutionary agent-based simulation and draws on some of the ideas mentioned above. In this section, we state the details of our model (see Algorithm 1 for a pseudocode formulation and the S1 Appendix for the ODD protocol [43, 44]).

**Algorithm 1:** Evolutionary simulation of the dynamics of well-being.

1: **for** generation *g* = 1 to *numGenerations* **do**	
2: **for** actionTime *t* = 1 to *numDays* **do**	▷ [LIFE CYCLE]
3: **for** agent *k* = 1 to *numAgents* **do**	
4: Compute motivation *M*_*k*_	▷ Eq 4
5: **if** *M*_*k*_ > *θ*_*k*_ **then**	▷ *θ* is the threshold for action; Eq 5
6: *k* chooses and executes aggressive action	
7: **else**	
8: *k* chooses and executes conservative action	
9: **end if**	
10: Determine the outcome	▷ food / no food
11: Update the food reward component, ${}_{f}H_{k}^{t}$	▷ Eq 7
12: Update the social component, ${}_{s}H_{k}^{t}$	▷ Eq 8
13: Update $E_{k}^{t}$	▷ Eq 9
14: **end for**	
15: **end for**	
16: **for** agent *k* = 1 to *numAgents* **do**	▷ [END OF LIFE / PROPAGATION]
17: **if** fitness $F_{k}$ is in the top 50% **then**	▷ Eq 10
18: *k* produces offspring with the same traits	
19: **end if**	
20: *k* terminates	
21: **end for**	
22: **end for**	

### The individual agent: motivation, action, rewards, and well-being

**Fig 1:**
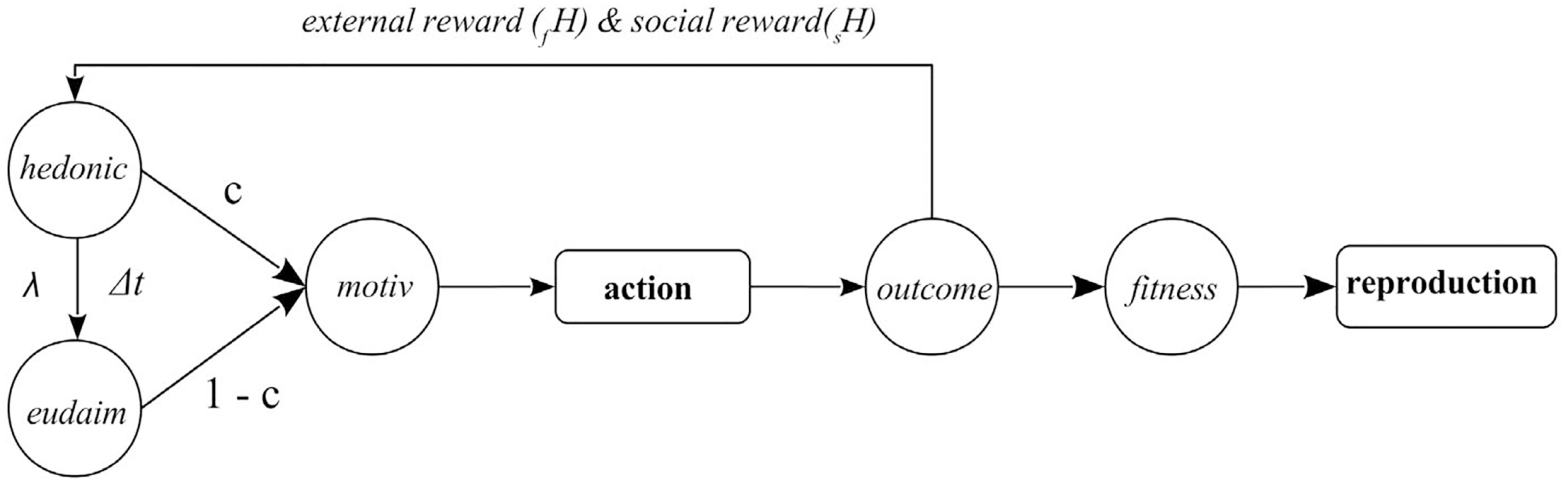
An agent’s basic action loop. Motivation prompts actions, which lead to outcomes. Outcomes reap external hedonic rewards (food-related _*f*_ *H*, and social _*s*_ *H*) and affect reproductive fitness. Hedonic states influence motivation, both directly, with the weight *c*, and through longer-term (“eudaimonic”) well-being *E*, via the weight 1 − *c*. The parameters Δ*t* and *λ* control, respectively, the time window over which *E* is estimated and the relative contributions of positive and negative changes of *H* (see Eq 9). After a set number of action cycles, each agent in the top half of the fitness distribution is allowed to produce offspring, which form the next generation; agents that belong to the current generation are terminated.

We simulate a population of foraging agents, each of which operates according to the action-outcome-valuation cycle illustrated in Fig 1 (for a more elaborate conception of what the block diagram of an autonomous agent could look like, see [45]).

The motivation *M* of agent *k* is a weighted sum of its hedonic and eudaimonic well-being, *H* and *E*, with the parameter 0 ≤ *c* ≤ 1 controlling their relative direct contributions; note that *H* also contributes to *M* indirectly, via its effect on *E* (see below):

$$\begin{array}{c} \begin{array}{c} M_{k}=c\cdot H_{k}+\left(1-c\right)\cdot E_{k} \end{array} \end{array}$$ (4)

The effect of motivation is controlled by a threshold *θ*, which depends on the agent’s past motivation:

$$\begin{array}{c} \begin{array}{c} \theta_{k}^{t}=\frac{1}{\sum_{j=1}^{t-1}\left(\gamma^{j}\right)}\sum_{i=1}^{t-1}\left(\gamma^{t-i}M_{k}\right) \end{array} \end{array}$$ (5)

where the threshold for agent *k* at time *t* is a weighted sum of past motivation values, *γ* ∈ [0, 1] being the forgetting factor, which gives more weight to recent motivations. When an agent’s motivation exceeds the threshold *θ*, it chooses a more aggressive action, by venturing farther away from its present location, in a random direction. If the motivation is below the threshold, the exploration range is shorter.

After carrying out the chosen action, the agent updates *H*, which consists of two components: _*f*_ *H*, based on finding food during exploration, and _*s*_ *H*, based on social comparison:

$$\begin{array}{c} \begin{array}{c} H_{k}=\left(1-s_{k}\right)\cdot_{f}H_{k}+s_{k}\cdot_{s}H_{k} \end{array} \end{array}$$ (6)

where *s*_*k*_, agent *k*’s sociality/food weighting parameter, is in the range of [0, 1]. The food-based component is computed as follows:

$$\begin{array}{c} \begin{array}{c} {}_{f}H_{k}^{t}{=}_{f}H_{k}^{t-1}+\alpha_{k}F_{k}^{t}-\beta_{f} \end{array} \end{array}$$ (7)

where *F* is the number of food units that the action yielded and *α*_*k*_ controls the contribution of food to agent *k*’s hedonic well-being; *β*_*f*_ is the food equivalent of the agents’ energy consumption per action cycle. For each agent, there is an upper bound on the maximum amount of food an agent can gain per cycle.

The social component of hedonic well-being _*s*_ *H* for agent *k* at time *t* is computed as shown below:

$$\begin{array}{c} \begin{array}{c} {}_{s}H_{k}^{t}=H_{k}^{t}-\frac{1}{n_{k}}\sum_{j=1}^{n_{k}}\left(H_{j}^{t}\right)+\beta_{s} \end{array} \end{array}$$ (8)

where the trait *n*_*k*_ represents the size of agent *k*’s social comparison group. Intuitively, an agent is happier when it is doing better than group average and less happy otherwise. *β*_*s*_ is the base hedonic well-being gain from socialization.

The agent’s eudaimonic well-being *E* is then computed from its present value of *H*, the memory of the past values of *H* extending over a number of cycles, and the rates of rise and fall of *H*. Specifically,

$$\begin{array}{c} \begin{array}{c} E_{k}^{t}=\frac{1}{\Delta t_{k}}\sum_{i=t-\Delta t_{k}}^{t}\left(H_{k}^{i}+s_{p}\left(\frac{dH_{k}^{i}}{di}\right)+s_{n}\left(\frac{dH_{k}^{i}}{di}\right)\right)\;\;\;\;\;\;\;\;\\ s_{p}\left(x\right)=\left\{\begin{array}{cc} {}_{p}\lambda_{k}\cdot x & x\geq 0\\ 0 & x<0 \end{array}\right.\;\;\;\;\;\;s_{n}\left(x\right)=\left\{\begin{array}{cc} 0 & x\geq 0\\ {}_{n}\lambda_{k}\cdot x & x<0 \end{array}\right.\;\; \end{array} \end{array}$$ (9)

where Δ*t*_*k*_ is the extent of the memory window for agent *k*. The step function *s*_*p*_ selects the weight assigned to upswings of *H* and and *s*_*n*_ — to downswings; _*p*_*λ*_*k*_ and _*n*_*λ*_*k*_ are the respective weights. Thus, every individual can in principle value positive and negative events differently.

In parallel with computing *H* and *E* (which are subsequently fed back and used to determine motivation, as per Eq 4), the agent’s fitness is updated, as follows:

$$\begin{array}{c} F_{k}^{t}=\sum_{i=1}^{t}\left(F_{k}^{i}-A_{k}^{i}-\beta_{0}\right) \end{array}$$ (10)

Thus, the fitness F of agent *k* at time *t* is the total amount of food $F_{k}^{t}$ that agent *k* consumed, less the cost $A_{k}^{t}$ of its actions (with aggressive and conservative actions weighted appropriately) and a base metabolic expenditure *β*_0_.

At the end of each generation, agents within the top 50% of the fitness distribution reproduce, passing their traits to their offspring, whose number is proportional to the parent’s fitness. All agents in the current generation are then terminated.

### The simulated environment

**Fig 2:**
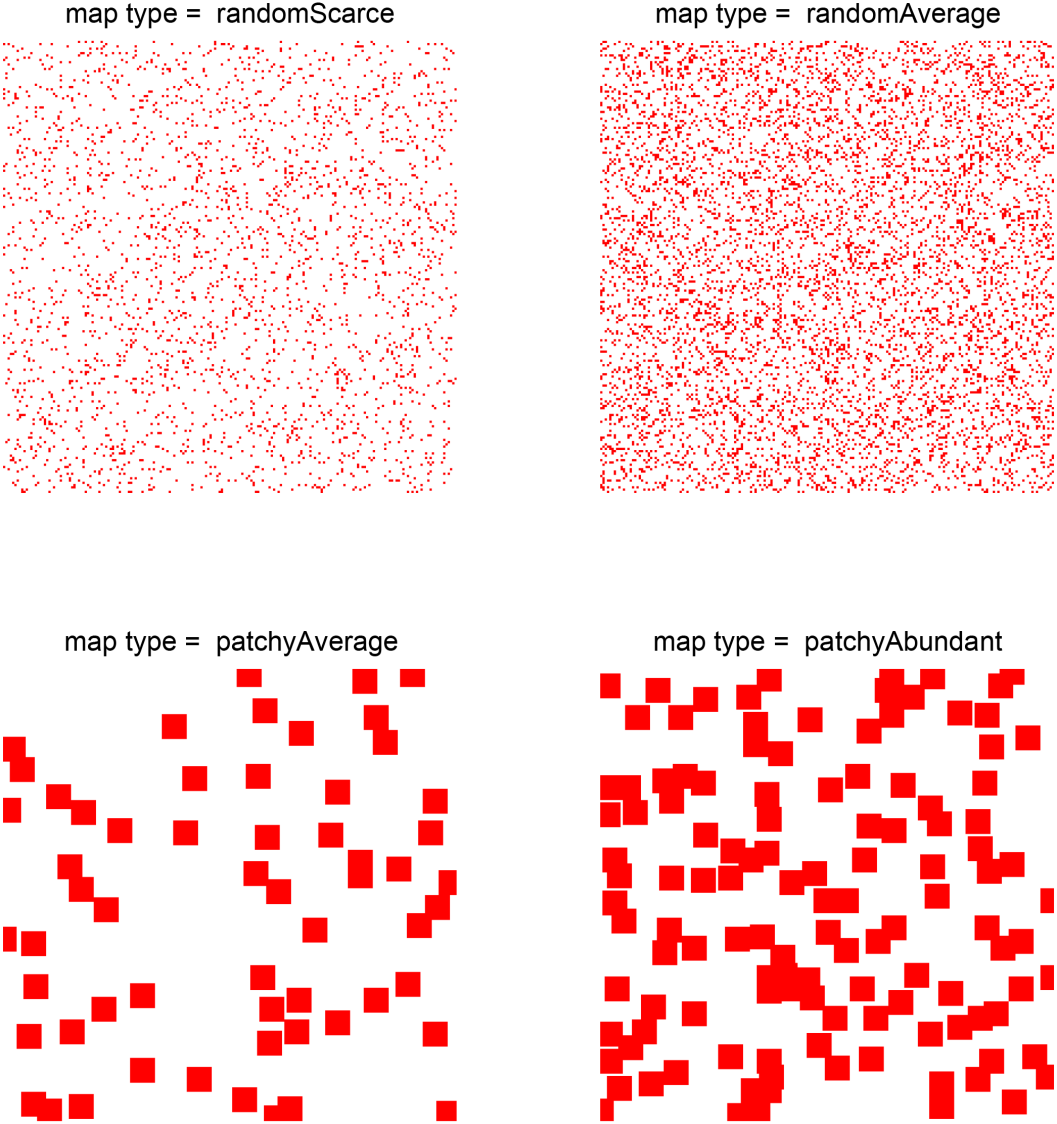
The simulations described in this paper were conducted for each of the four types of food distribution shown here: random scarce, random average, patchy average, and patchy abundant.

We simulated four types of environment, which differed in the spatial distribution of food, as illustrated in Fig 2: random scarce (top left); random average (top right); patchy average (bottom left); and patchy abundant (bottom right). Each 200×200 environment was populated in every generation by 400 agents. At the end of every generation cycle, the environments were initialized anew with their corresponding food distributions.

**Fig 3:**
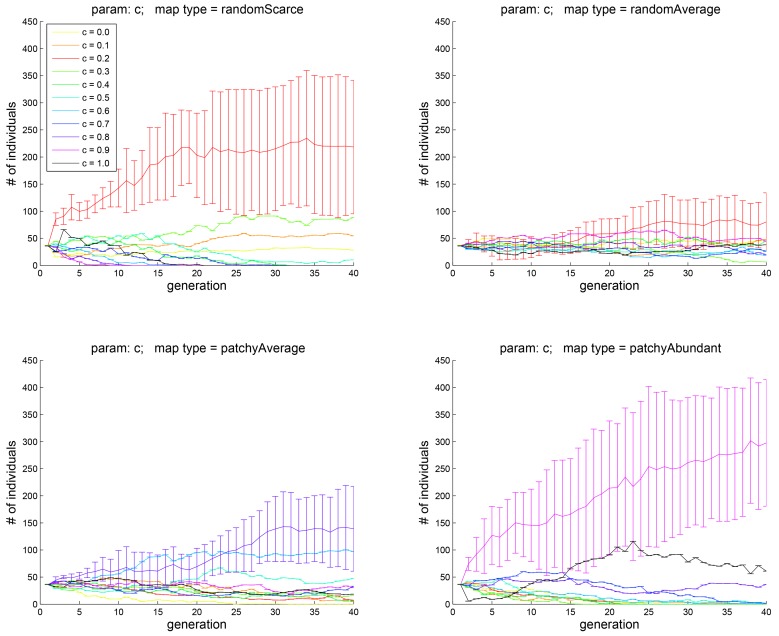
Experiment 1A: the effect of the parameter *c*, which controls the relative contributions to motivation of the hedonic well-being *H* and eudaimonic well-being *E* (see Eq 4). In each simulation, the initial population consistent of 11 cohorts, over which the value of *c* ranged from 0 to 1 in increments of 0.1. The four subplots correspond to the four different map types shown in Fig 2. The abscissa shows the generation number; the ordinate is the number of individuals in each cohort. See text for interpretation.

**Fig 4:**
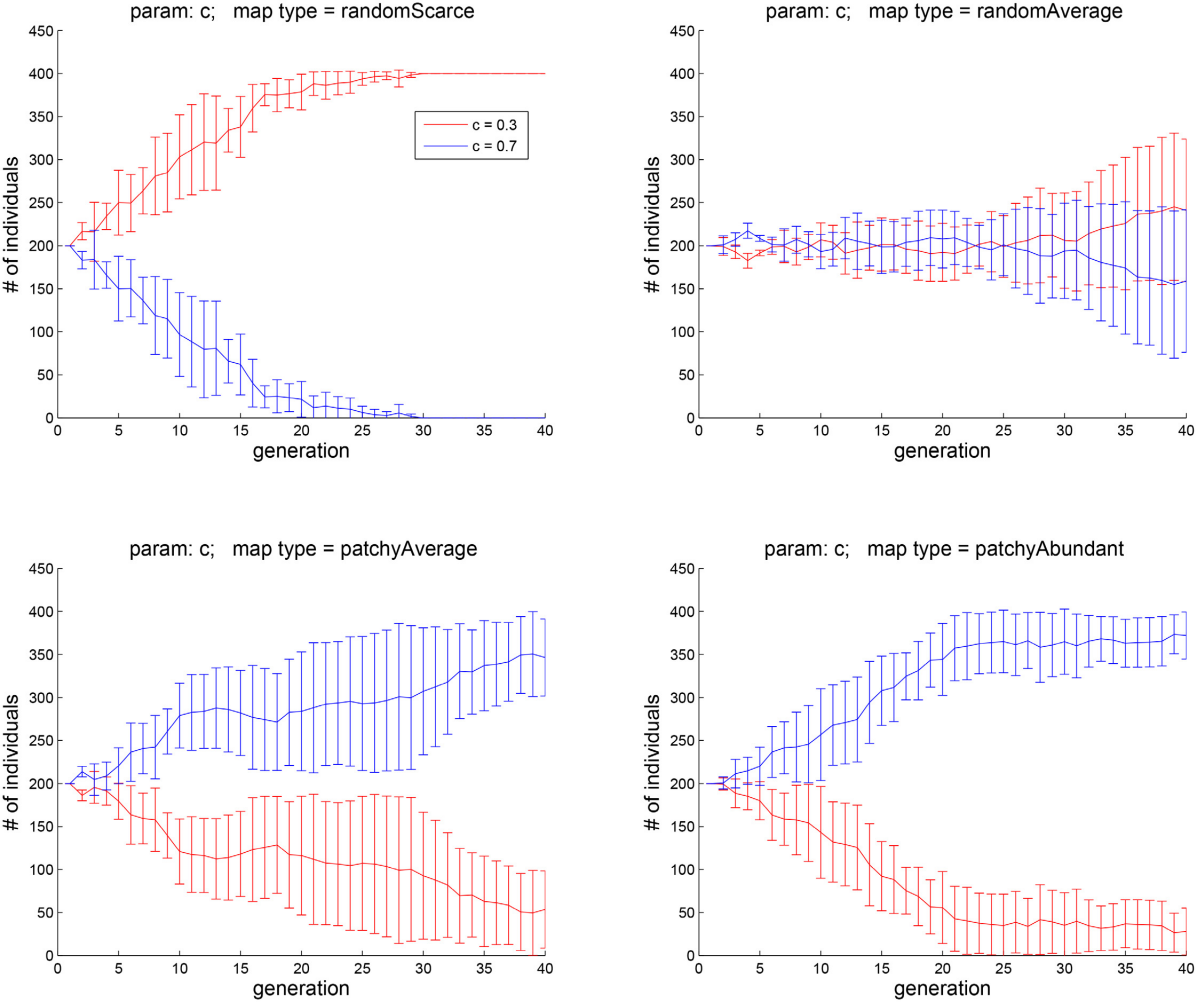
Experiment 1B: here, subpopulations with two values of *c*, 0.3 and 0.7, were pitched against each other. The lower value, corresponding to immediate or hedonic well-being *H* contributing less to motivation, appears to be advantageous only in the scarce-food environment. The higher value of *c*, is more advantageous when food is patchy and not rare to find.

**Fig 5:**
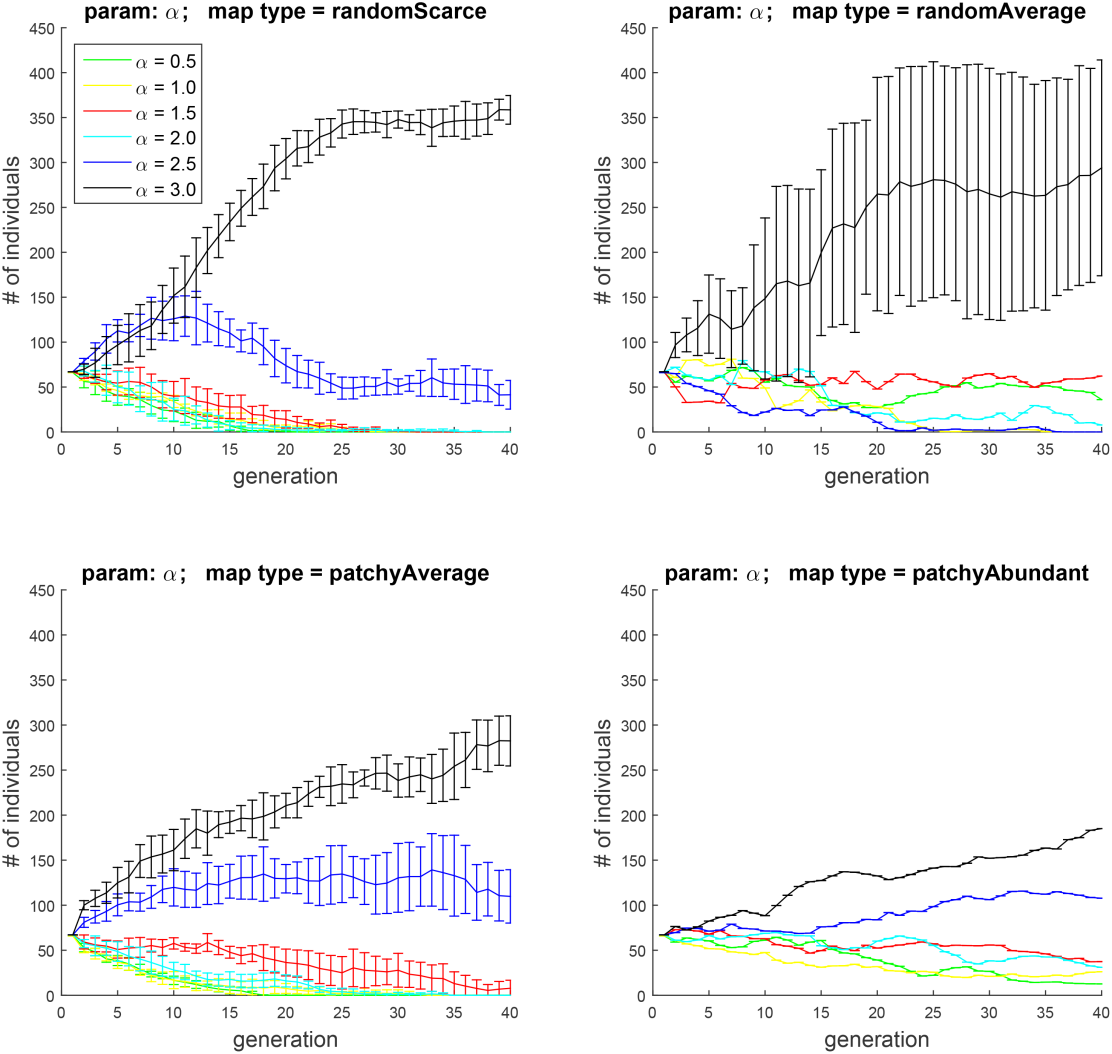
Experiment 2A: the effect of the parameter *α*, which sets the contribution of food rewards to *H*. As before, the plots show, for each successive generation, the mean cohort sizes and 95% confidence intervals over 10 runs. The results indicate that letting food contribute more strongly to hedonic well-being, and hence to motivation, is more advantageous under conditions of food scarcity.

**Fig 6:**
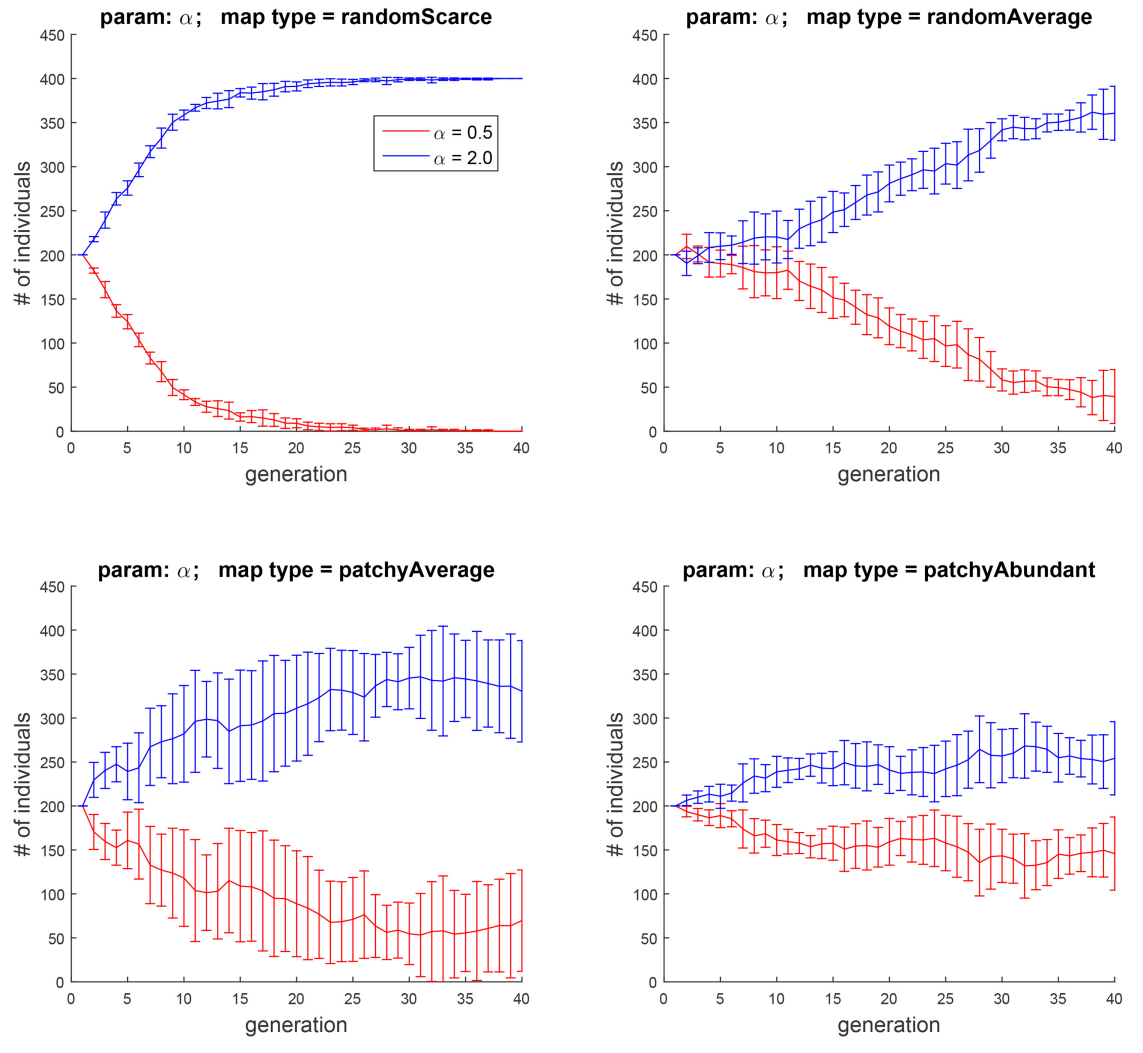
Experiment 2B: the effect of the contribution of food rewards to *H*, when two subpopulations with *α* = 0.5 and *α* = 2.0 pitched against each other. As in Fig 5, the effect is more pronounced when food is food is not abundant.

**Fig 7:**
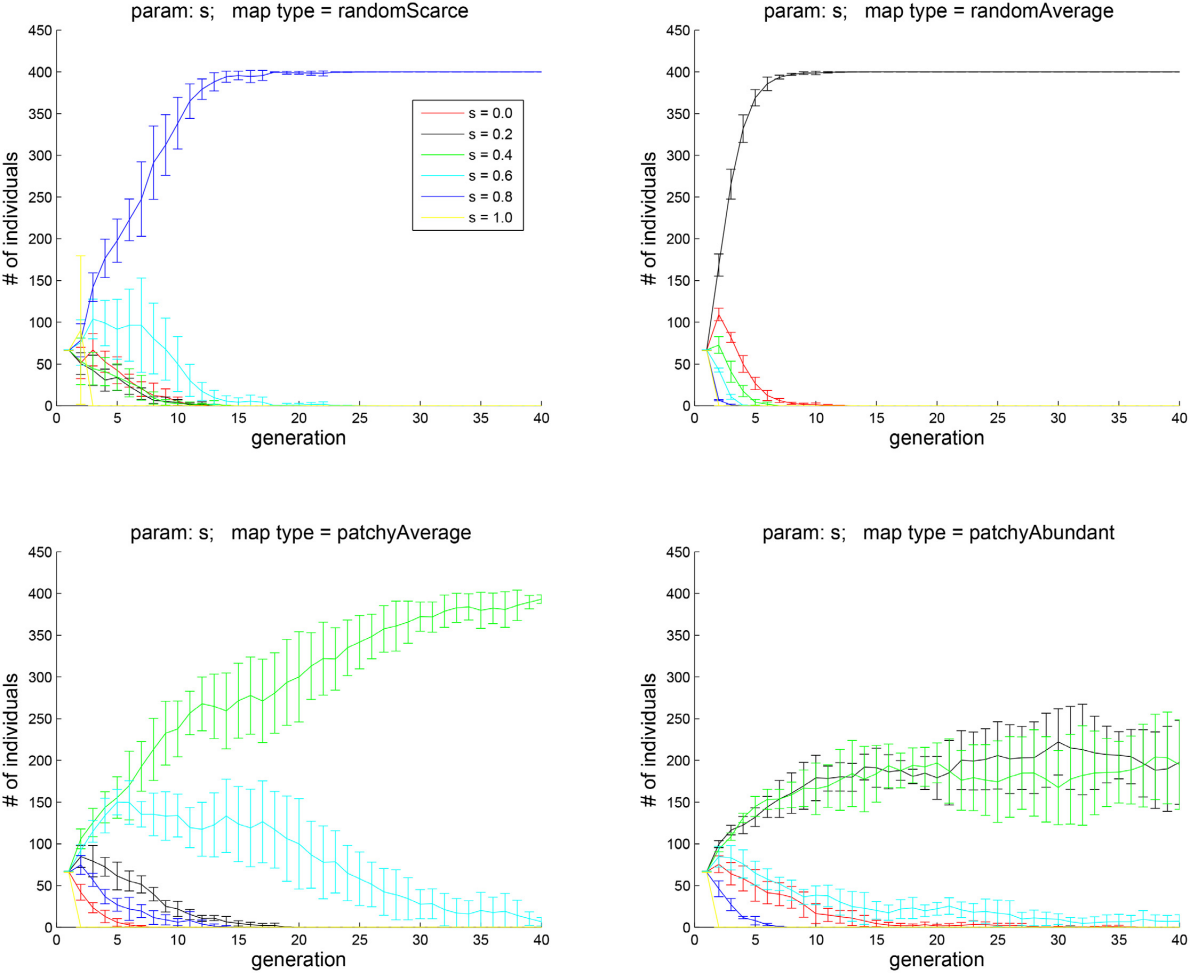
Experiment 3A: the effect of an agent’s social preference. The four subplots correspond to the four environment types shown in Fig 2. Social competitiveness (allowing one’s social group *H* to drag down one’s own happiness) is shown to play an important role. When food is abundant, agents weight more on foraging is more successful. When food is scarce, agents weight more on socialization emerge as more successful.

**Fig 8:**
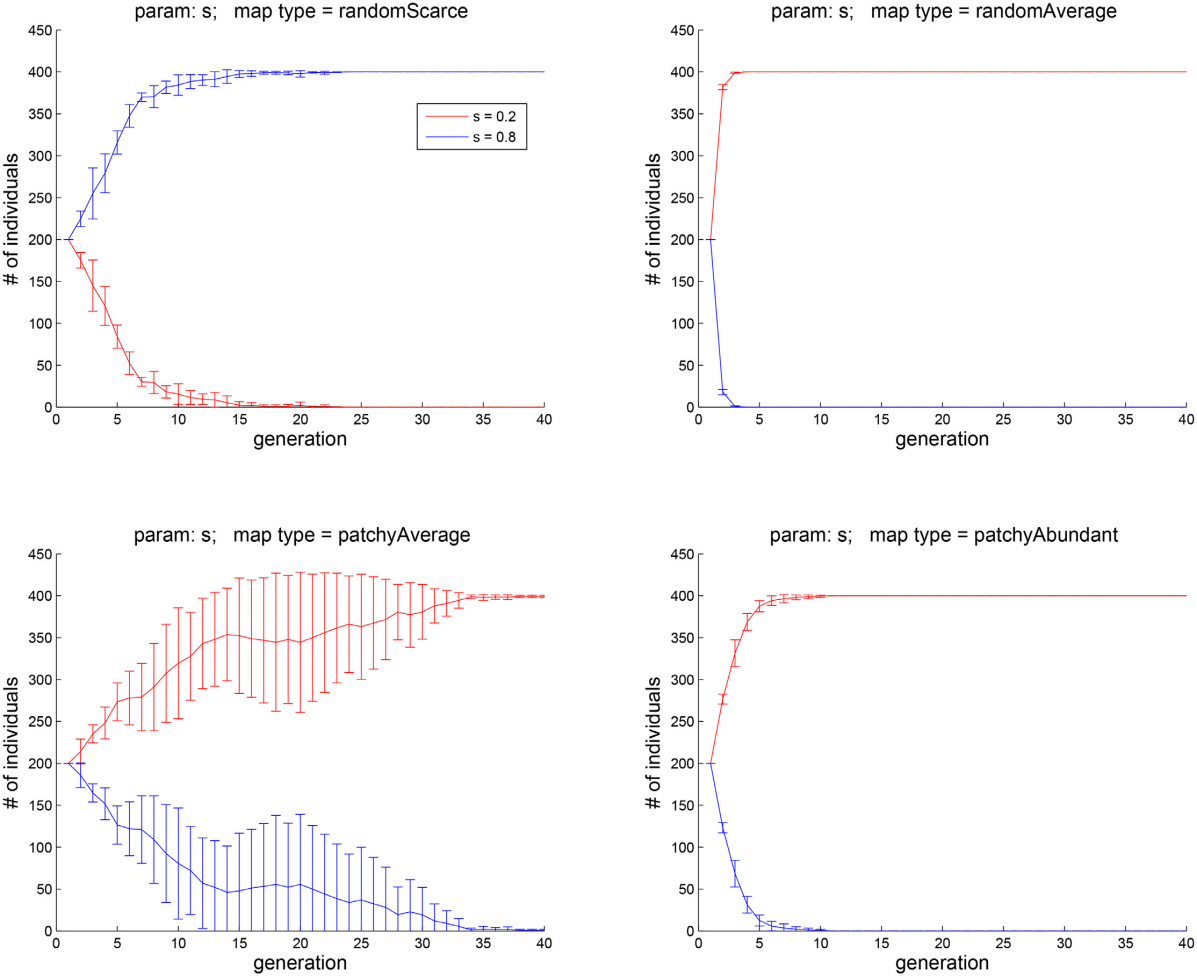
Experiment 3B: subpopulations with two values of *s*, 0.2 and 0.8, were pitched against each other. The higher value of *s*, corresponding to social comparison contributing more towards *H*, appears to be advantageous only in the scarce-food environment. The lower value of *s* is more advantageous when food is average or abundant.

**Fig 9:**
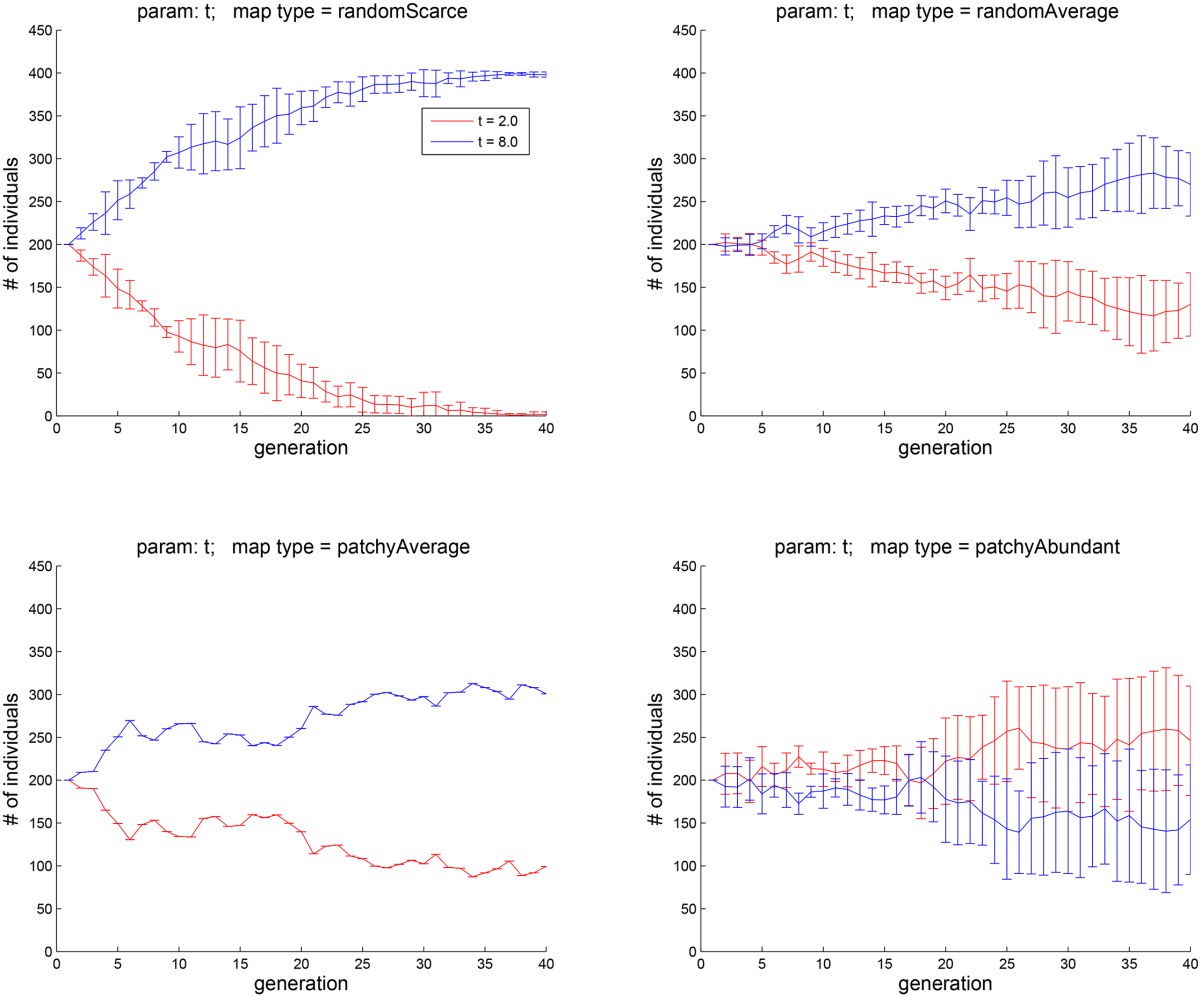
Experiment 4A: the influence of memory duration. The four plots corresponds to the four map types shown in Fig 2. When resources are scarce, agents with longer memory of past hedonic states tend to do better. When resources are abundant, this effect disappears.

**Fig 10:**
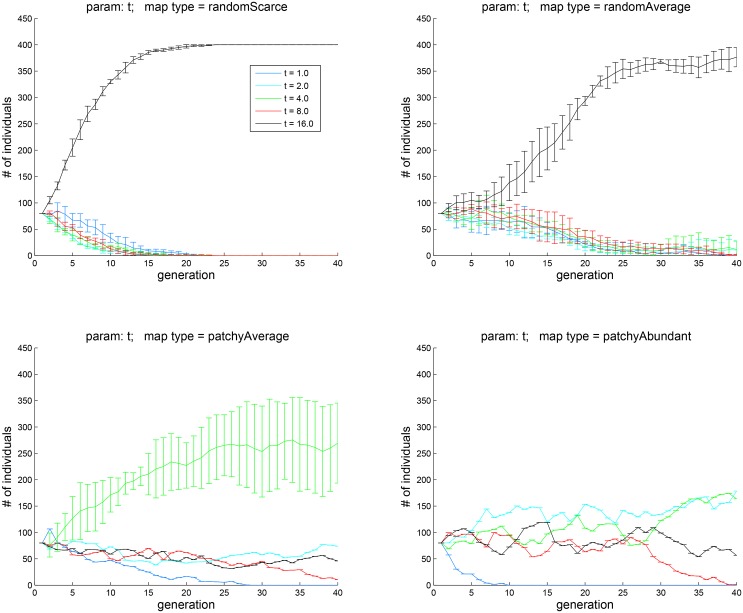
Experiment 4B: pitching agents with Δ*t* = 2 against those with Δ*t* = 8. The four plots corresponds to the four map types shown in Fig 2. The results are similar to those in Exp. 4A.

**Fig 11:**
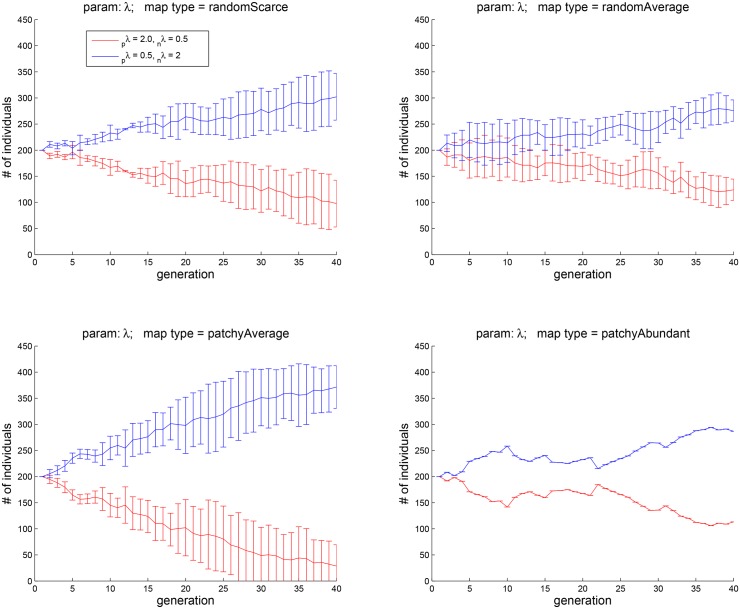
Experiment 5: the dependence of eudaimonic well-being on the rise and fall of hedonic states. The four plots correspond to the four map types shown in Fig 2. Agents with a more positive outlook (_*p*_*λ* = 2, _*p*_*λ* = 0.5) tend to do better in all four types of environments.

**Fig 12:**
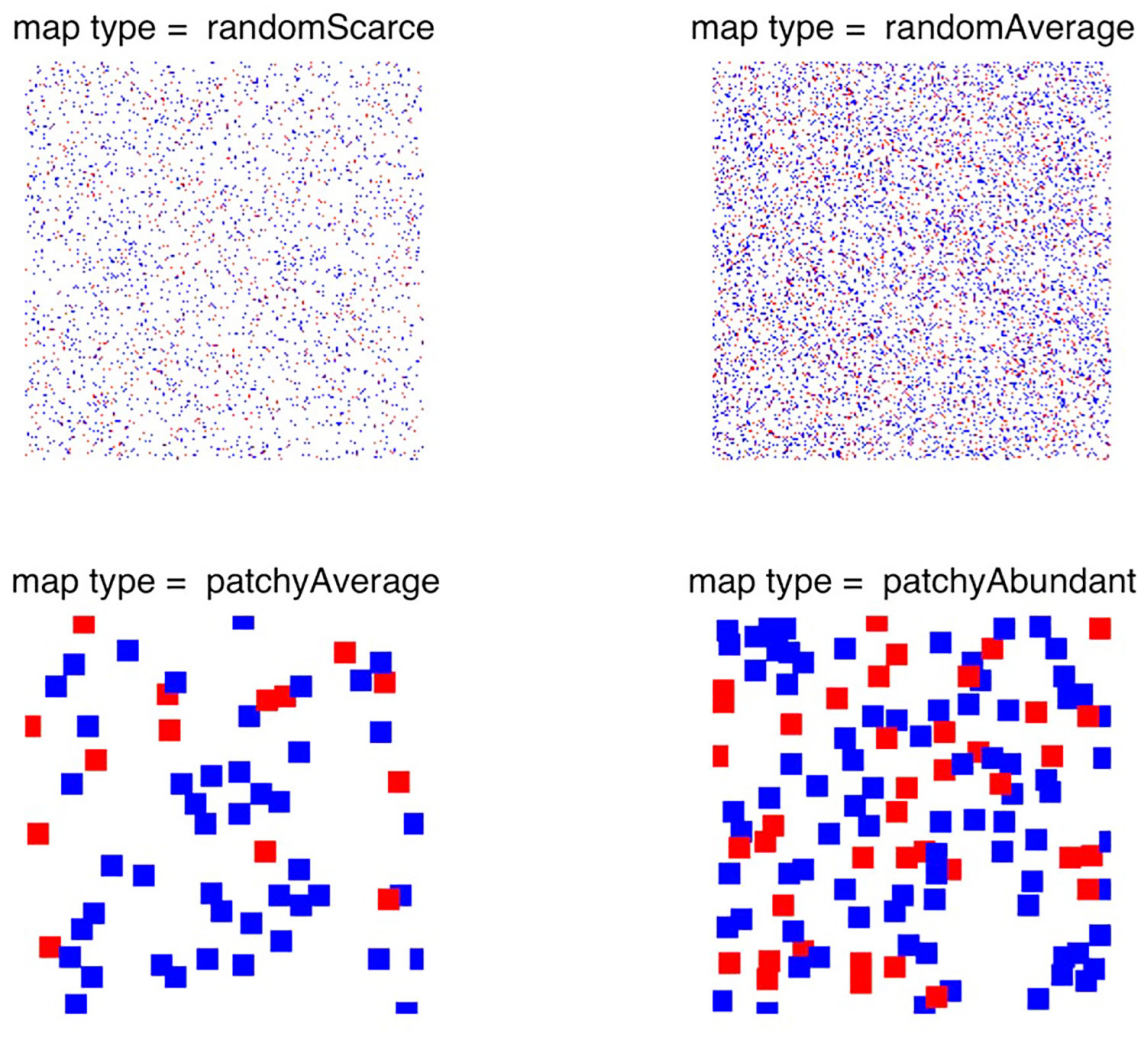
The simulations in experiment 6 were conducted for each of the four types of food distribution shown above, with blue representing poison and red representing food resources.

**Fig 13:**
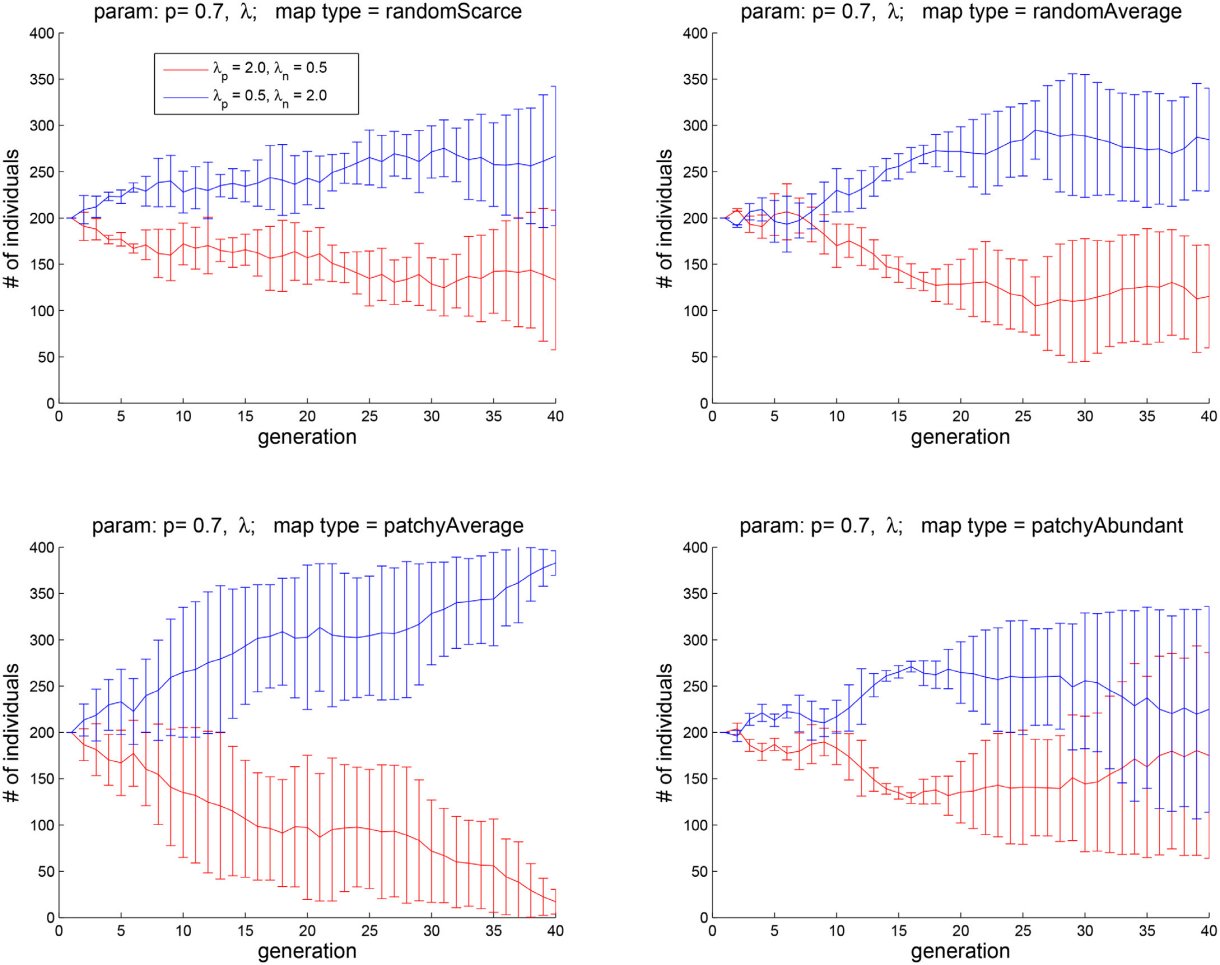
Experiment 6: the dependence of eudaimonic well-being on the rise and fall of hedonic states in the environments that contain both food and poison. The four plots correspond to the four map types shown in Fig 12 Agents with a more negative outlook (_*p*_*λ* = 2, _*p*_*λ* = 0.5) tend to do better in all four types of environments.

In each of the experiments described below, the parameter (trait) of interest was discretized as appropriate, so that equal proportions of the population carried each level of the trait; the other parameters were kept fixed. Each experiment was repeated 10 times; each of the Figs 3–13 below shows the means and the 95% confidence intervals for the number of carriers in the population of each level of the trait of interest, plotted against generation number.

The implementation of our ABM model and the code for the experiments are available at https://www.openabm.org/model/4934. In addition, the details of the implementation are described in the S1 Appendix, which follows the ODD (Overview, Design concepts, and Details) protocol [43, 44].

## Experiment 1: the effect of relative contributions of *H* and *E* to motivation

In this experiment, we explored the effect of the parameter *c*, which, as per Eq 4, determines the relative contributions of the agent’s hedonic (*H*) and eudaimonic (*E*) well-being to its motivation (cf. Fig 1). Specifically, for values of *c* that are close to 1, the dominant contribution is that of *H* and the agent is motivated primarily by immediate external outcomes of its actions (that is, food or social rewards). In comparison, for values of *c* that are close to 0, the longer-term well-being *E* dominates.

To estimate the effect of *c* over its entire range of [0, 1], we first stepped its value from 0.0 by 0.1, resulting in 11 cohorts of 400/11 ≈ 36 agents each, which had the same value of *c* in the first generation. Fig 3 shows how the number of agents in each cohort evolved over successive generations; the four panels correspond to the four different map types shown in Fig 2. The data for each panel were generated by repeating the experiment 10 times with same parameter settings; the points and error bars show the means and 95% confidence intervals. Different colors represent different value of *c* as indicated in the legend.

Because at the end of each generation, only the agents with the higher fitness are allowed to reproduce, the number of agents with different values of the trait *c* changes over evolutionary time. After about 40 generations, the population sizes begin to stabilize. As Fig 3, top left, suggests, when the food is dispersed and scarce, agents with *c* = 0.2 predominate. When food is no longer scarce, agents with this value of trait *c* still have a higher mean population than the rest. When food distribution is patchy, a higher value of *c* = 0.8 — that is, more weight given to immediate well-being *H* — is more advantageous. When food is abundant (bottom right), agents with *c* = 0.9 are much more dominant after 40 generation cycles.

To focus on the contrast between lower and higher values of *c*, we repeated this experiment with an initially dichotomous population in which half of the agents had *c* = 0.3 and the other half *c* = 0.7. The results are shown in Fig 4. The fates of agents carrying each of the two traits are now clearly divergent in each of the four environment types. Specifically, when food is random and scarce (top left), having the lower value of *c* — that is, being motivated more by longer- than by shorter-term well being — is advantageous. When food is random and no longer scarce (top right), there is no clear advantage to either value of *c*. In the remaining two environment types (bottom panels), agents with the higher value of *c* dominate. Thus, the evolutionary performance of agents with different contributions of hedonic and eudaimonic well-being to motivation depends on the environment type and resource distribution.

## Experiment 2: the effect of the contribution of food to *H*

In this experiment, we investigated the effects of the amount of hedonic boost that the agent gets from each encounter with food, controlled by the parameter *α* in Eq 7. To that end, we first explored the general effect of this parameter, by running simulations with six initial cohorts, created by discretizing *α* into six values in the range of [0.5, 3] with a step size of 0.5.

The results appear in Fig 5. Not surprisingly, in an environment where food is random and scarce (top left), assigning food a larger weight is advantageous. In comparison, when food is abundant (bottom right), this advantage is smaller. Pitching cohorts with *α* = 0.5 and *α* = 2 against each other (Fig 6) offers a starker contrast between the evolution of the two populations. The direction of the effect is, however, the same as before.

## Experiment 3: the effect of preference of exploration and socialization as a factor in *H*

The social-competitive aspect of well-being is simulated in our experiments by letting the average hedonic well-being *H* of the agent’s friends contribute negatively to its own hedonic well-being. As per Eq 6, agent *k*’s hedonic well-being *H* depends on trait *s*_*k*_, which determines the relative contribution of socialization and exploration. In terms of the social hedonic well-being, the assumption is that an agent is happier when its *H* is above the average of its comparison group and less happy when it is below the average. For each agent, there is an individual parameter that controls how much weight it assigns to food vs. social comparison. Agent *k*’s social comparison group is determined at the outset by randomly choosing *n*_*k*_ other agents, regardless of their positions on the map. The agent then maintains the same social circle during its lifetime. In the following experiment, the size of social circle *n*_*k*_ was set to 8 for all agents.

Fig 7 shows the effect of the parameter *s*_*k*_ that controls how much social comparison contributes to *H*. After 40 generations, the population sizes begin to stabilize. As Fig 7, top left, suggests, when food is dispersed and scarce, agents with *s* = 0.8 dominate. When food is more abundant, the advantage of the trait of having social comparison contribute more towards *H* is diminished. When food distribution is patchy and abundant (bottom right), agents with *s* = 0.2 and *s* = 0.4 dominate — that is, agents that are less prone to social comparison perform better when resources in the environment are plentiful.

To focus on the contrast between lower and higher values of *s*, we repeated this experiment with an initial population in which half of the agents had *s* = 0.2 and the other half *s* = 0.8. The results are shown in Fig 8. The fates of agents carrying each of the two traits are now clearly divergent in each of the four environment types. Specifically, when food is random and scarce (top left), having the higher value of *s* — that is, being more socially aware — is advantageous. When food is patchy and no longer scarce (bottom left), having a lower value of *s* is more advantageous. Thus, the overall effect of social comparison seems to be to promote success in harsh environments (at least for the chosen settings of the other relevant parameters, notably, the social circle size *n*_*k*_, for which a range of values around 8 was explored, with similar results).

## Experiment 4: the effect of the timing of the contribution of *H* to *E*

This experiment and the next one (described in the following section) focus on the parameters that control the dynamics of the contribution of hedonic well-being *H* to eudaimonic well-being *E*. In experiment 4, we studied the effect of the duration of the temporal window over which *H* is accumulated before contributing to *E* — the parameter Δ*t* in Eq 9. The larger Δ*t*, the stronger the smoothing effect that *E* exerts over *H* as they jointly influence motivation.

The results of Experiment 4A, in which five cohorts with different values of Δ*t* (1, 2, 4, 8, 16) were tested, are shown in Fig 9. The four panels correspond to the four environment types of Fig 2. As the top left panel shows, when food is scarce, agents that have a longer memory of hedonic states (Δ*t* = 16) do better. Agents with this trait also do better in other map types where food is random-average. However, as more food resources appears on the map, it takes more generation cycles for this trait to dominate. When food is random-abundant, it no longer does better.

In Experiment 4B, the population consisted of two cohorts, one with Δ*t* = 2 and the other with Δ*t* = 8. The results appear in Fig 10. The fates of agents carrying each of the two traits are now clearly divergent in each of the four environment types. Specifically, when food is random and scarce (top left), having a longer memory of past hedonic well-being (Δ*t* = 8) is advantageous. When food is average (top right and bottom left), having a higher value of Δ*t* is still advantageous. However, when food is abundant (bottom right), agents with a smaller Δ*t* do better.

## Experiment 5: the relative influence of positive and negative changes in *H* on *E*

In Experiment 5, we investigated the effect of balancing the contributions to *E* of positive and negative changes in *H*. In Eq 9 these contributions are represented by the possibly different weights, _*p*_*λ* and _*n*_*λ*, assigned to the time derivative of *H*. Intuitively, the eudaimonic (“life evaluation”) state of an agent with a negative outlook is affected more strongly by a drop than by a rise in *H* (_*p*_*λ* > _*n*_ *λ*); for an agent with a positive outlook, the relationship is opposite (cf. the computational formulation of optimism and pessimism in [46]).

Fig 11 shows the results of pitching against each other two cohorts, one with _*p*_*λ* = 2.0, _*n*_ *λ* = 0.5 and the other with _*p*_*λ* = 0.5, _*n*_ *λ* = 2.0. The four plots correspond to the four map types of Fig 2. It is interesting to observe that having a positive outlook has an advantage in all four types of environment that we have tested. This finding may be compared to the positive-mood bias that characterizes the general human population and to the evolutionary accounts that have been offered for this bias [21, 47].

## Experiment 6: the relative influence of positive and negative changes in *H* on *E*, in the presence of negative events

In natural foraging environments, agents typically experience both positive events (e.g., encounters with food) and negative ones (e.g., encounters with noxious or poisonous items). In this experiment, we investigated how agents perform in an environment that contains both positive and negative events. Specifically, we focus on the contributions to *E* of positive and negative changes in *H* in such environments.

To characterize the relative prevalence of the two types of events, we introduce a variable *p*, in the range of [0, 1], which controls the proportion of negative (“poison”) items on the map. In this experiment, we used maps with *p* = 0.7, illustrated in Fig 12. The agent’s fitness is assumed to decrease by one unit when a poison item is consumed. When *p* is small, its influence on the convergence of parameters _*p*_*λ* and _*n*_*λ* that control the weight of the rise and fall of hedonic well-being is minimal. For instance, when we set *p* = 0.1, the pairwise comparison of _*p*_*λ* and _*n*_*λ* yielded results that were very similar to those in Fig 11 in experiment 5.

Fig 13 shows the results of pitching against each other the same two cohorts that participated in Experiment 6: one with _*p*_*λ* = 2.0, _*n*_ *λ* = 0.5 and the other with _*p*_*λ* = 0.5, _*n*_ *λ* = 2.0, in environments with *p* = 0.7. The four plots correspond to the four map types of Fig 12. It is interesting to observe that having a negative outlook has an advantage in all four types of environments. This finding suggests that in harsh environments agents with a conservative outlook (larger _*n*_*λ*) have higher fitness.

## Summary and Discussion

The evolutionary experiments described in this paper operationalized the happiness of an agent as a pair of state variables — momentary, *H* (for hedonic), and cumulative, *E* (for eudaimonic) — that jointly help regulate behavior by mediating between outcomes and motivation. The agent’s behavior and interaction with the environment arose from the dynamics of the control loop (motivation to action to outcome and back via happiness to motivation; Fig 1), along with a handful of parameters. Lifetime outcomes accrued to form fitness, which in turn determined how many, if any, of the agent’s clones became part of the next generation.

As an exercise in simulated evolution, this setup is limited in many respects. In particular, the agents’ parameters were fixed, making them capable only of what Dennett called “learning by death” [48]. This necessitated a manual search for a viable combination of parameters (so as to avoid unprovoked mass extinctions) prior to running the actual experiments. This, however, turned out to be not too difficult, which suggests that our results are relatively general. More importantly, our working hypothesis regarding the nature of happiness and its role in behavioral control proved fruitful in that it yielded unambiguous and intuitively interpretable results. Stated concisely, our main findings are as follows:

Exp.1 *The effects of the relative contributions of H and E to motivation:* When food is scarce, agents that are motivated more by eudaimonic (longer-term) well-being *E* than by hedonic (momentary) well-being *H* dominate. When food is abundant, agents that are motivated more by *H* than by *E* dominate.
Exp.2 *The effects of the contribution of food to H:* Agents that get a larger hedonic boost from finding food dominate, to a degree that depends on food distribution.
Exp.3 *The effects of the size of social comparison circle:* When food is scarce, agents that give more weight to a comparison of their outcomes with those of their “friends” do better. When food is abundant, this effect is reversed.
Exp.4 *The effects of memory for past H values:* When food is scarce, agents that integrate their hedonic states over a longer time window dominate. When food is abundant, the advantage of having longer memory dissipates.
Exp.5 *The effects of the differential contribution of positive and negative changes in H:* Agents with a more positive outlook, which attach more importance to upswings in *H* than to downswings, dominate in all four types of environment.
Exp.6 *The effects of the differential contribution of positive and negative changes in H in environments with both positive and negative events:* In relatively harsh mixed-valence environments, agents with a more conservative outlook (larger _*n*_*λ*) have higher fitness.

The general lesson from these findings is that the mix of happiness-related behavioral control parameters that works the best, in that agents that are endowed with it come to dominate the population, depends on the environment. In reality, environmental conditions (such as the amount and the distribution of food) are subject to change, typically on multiple time scales. It would be interesting, therefore, to see what happiness and happiness-tuning traits emerge under various schedules of environmental stress. Note that coping with environmental changes does not necessarily require learning in the phenotype [49], although such an ability (as in, for instance, reinforcement learning [21, 36]) may make for smarter and more efficient agents [48].

A more specific lesson that can be drawn from our results is that the effects of attaching more weight to longer-term than to momentary happiness and of extending the memory for past happiness are both stronger in an environment where food is scarce. Furthermore, in such an environment the “arms race” of relative consumption [8, 9], in which the agent’s well-being is diminished if its neighbors are also well or better off, is more detrimental to survival. Finally, we saw that agents with a positive outlook, whose longer-term happiness gets more increase from positive events than decrease from negative ones, is generally advantageous, except in particularly harsh environments.

On a normative-philosophical note, this set of findings may be loosely compared to the sentiment expressed in Laozi’s *Dao De Jing* under the heading of curbing desire: “The satisfaction of contentment is an everlasting competence” [50]. It may indeed be advisable, at least under conditions of scarcity or adversity, to focus on longer-term well-being or eudaimonia (“contentment”) over momentary pleasures and to be less envious of one’s neighbors; also, in general, to mark happy events more than unhappy ones.

To make the parallels between evolutionary agent-based simulations findings and psychological (let alone philosophical) works on happiness somewhat less strained, a number of extensions to the present work can be undertaken. Specifically, the studies reported here should be repeated with more realistic agents, endowed with evolvable genotypes and capable of reinforcement learning (e.g., [37]). Such agents should then be faced with changing environments: it would be interesting to see whether mechanisms can evolve that not only use happiness for dynamically controlling actions, but also control the dynamics of happiness in response to environmental and other stress. On the basis of previously offered arguments regarding the importance of “learning the world” [51], we conjecture that agents capable of intrinsically motivated model-based reinforcement learning in particular would, like people, attach no less value to the pursuit of happiness than to its attainment [7].

## Supporting Information

S1 Appendix The supporting information follows the ODD protocol. In the document, more details are provided such as the agents’ initialization parameters and their values or ranges of values. In addition, the values of the key parameters used in each experiment are presented.
(PDF)
