# Supplementary material for: Between Pleasure and Contentment: Evolutionary Dynamics of Some Possible Parameters of Happiness
Source: PLoS One. 2016 May 4;11(5):e0153193. doi: 10.1371/journal.pone.0153193 (PMC4856412; doi:10.1371/journal.pone.0153193)
Supplement: S1 Appendix — In the document, more details are provided such as the agents’ initialization parameters and their values or ranges of values. In addition, the values of the key parameters used in each experiment are presented. (PDF) [file pone.0153193.s001.pdf]

Appendix: the ODD protocol for our ABM model

Overview

As per the ODD (Overview, Design concepts, and Details) protocol for the specification of agent-based models [43,44], we offer here the details of our ABM simulations; the code is available at `https://www.openabm.org/model/4934`.

Purpose

The purpose of our agent base simulation model (ABM) is to investigate, in an evolutionary setting, a series of questions pertaining to happiness. These include (i) the role of balancing momentary well-being against longer-term contentment (the "happiness of pursuit" [7]); (ii) the effects of drawing a contrast between oneself and one's social circle (what economists term "relative consumption" [8,9]); and (iii) the adaptive role of differential sensitivity to positive and negative turns in momentary well-being.

State variables and scales

The computation of this simulation comprises four components: environment initialization, agent initialization, agent behavior simulation, and evolutionary reproduction.

We simulated four types of environment, which differed in the spatial distribution of food, as illustrated in Fig. 1 in the main text: random scarce (top left); random average (top right); patchy average (bottom left); and patchy abundant (bottom right). Each 200×200 environment was populated in every generation by 400 agents. The food density for scarce environment is 1/15. The food density for average environment is 1/6, and for abundant environment is 1/3. For each map, the location of the 400 agents is randomly selected.

For agent behavior simulation, we implemented a foraging scenario, in which each agent operated according to the action-outcome-valuation cycle illustrated in Fig. 1 (for a more elaborate conception of what the block diagram of an autonomous agent could look like, see [45]). This figure describes an agent's basic action loop. Motivation prompts actions, which lead to outcomes. Outcomes reap external hedonic rewards (food-related ${}_fH$, and social ${}_sH$) and affect reproductive fitness. Hedonic states influence motivation, both directly, with the weight c, and through longer-term ("eudaimonic") well-being E, via the weight $1 - c$.

The evolutionary reproduction step determines which agent can reproduce and how many offspring it can have. After a set number of action cycles, each agent in the top half of the fitness distribution is allowed to produce offspring, which form the next generation; agents that belong to the current generation are terminated. The number of offspring an agent produces is proportional to its fitness rank. The higher the fitness, the larger the number of offspring that carries its traits (parameter settings).

Process overview and scheduling

The simulation consists of a series of generation cycles, each of which corresponds to the complete lifetime of a population of agents. During their lifetime, agents choose their

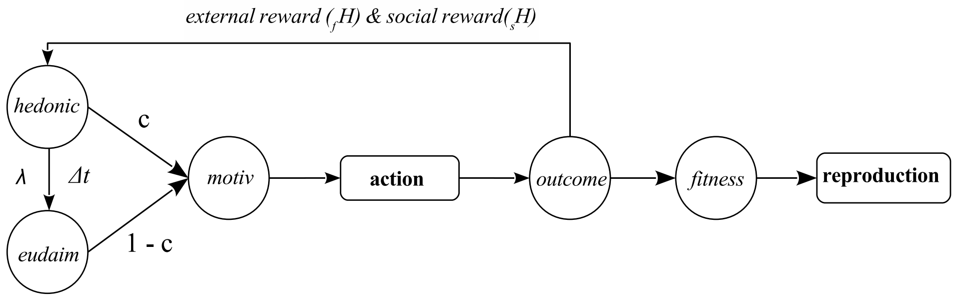

Figure 1. An agent's basic action loop. Motivation prompts actions, which lead to outcomes. Outcomes reap external hedonic rewards (food-related $_fH$, and social $_sH$) and affect reproductive fitness. Hedonic states influence motivation, both directly, with the weight c, and through longer-term ("eudaimonic") well-being E, via the weight $1 - c$. The parameters Δt and λ control, respectively, the time window over which E is estimated and the relative contributions of positive and negative changes of H (see eq. 6). After a set number of action cycles, each agent in the top half of the fitness distribution is allowed to produce offspring, which form the next generation; agents that belong to the current generation are terminated.

actions, experience outcomes, and accrue fitness. At the end of each generation cycle, agents in the top half of the fitness distribution are allowed to replicate.

Design concepts

Evolutionary convergence: population dynamics emerge from the behavior of the individual agents. Our model is based on evolutionary simulation. Initially, the number of agents with the same trait is the same. Over multiple generations, dominant traits emerge through evolutionary selection.

Motivation guided behaviors: behavior is considered to be motivated if it is at least partly determined by its expected consequences [2]. The control of motivated behavior involves internal states, which represent goals or expected outcomes. In our simulations, if an agent is strongly motivated, it tends to undertake more aggressive actions (e.g., explore farther afield). If it is less motivated (e.g., because of lower well-being), it tends to explore more conservatively.

Social interaction: in behavioral economics, it is well known that people's perceived conditions with regard to the so-called positional goods depend on those of their social circle or comparison group [8,9]. Intuitively, subjects can be more or less happy with the same absolute level of a positional good (say, a house of a given size), depending on the levels of their neighbors. The study of Baggio and Papyrakis [33] explored the effects of this type of social comparison. In our simulations too, social comparison is one of the factors in well-being.

Details

We now turn to the description of our own work, which uses evolutionary agent-based simulation and draws on some of the ideas mentioned above.

Parameter	Meaning	Value or Range
n_R	the height of the map	200
n_C	the width of the map	200
k	the index of the agent	$[1, 400]$
T	the agents' lifetime duration	50
G	the total number of generation cycles	40
H_k	the hedonic well-being of an agent k	200
E_k	the eudaimonic well-being for agent k	200
n_k	the size of agent k's social comparison circle	8

Table 1. Overview of the agents' initialization parameters and their values or ranges of values.

Initialization

The initialization of the parameters are described in Table 1. The parameters values as shown in the right column of the table.

Input

The input of our model includes the number of generation cycles, which is set to 40, and, for each generation, the number of steps, which is set to 50. The rest of the parameters are discretized according to the needs of each experiment.

The model

We simulate a population of foraging agents, each of which operates according to the action-outcome-valuation cycle illustrated in Fig. 1 (for a more elaborate conception of what the block diagram of an autonomous agent could look like, see [45]). The algorithm is described in the main text.

The motivation M of agent k is a weighted sum of its hedonic and eudaimonic well-being, H and E, with the parameter $0 \leq c \leq 1$ controlling their relative direct contributions; note that H also contributes to M indirectly, via its effect on E (see below):

$$M_k = c \cdot H_k + (1 - c) \cdot E_k \tag{1}$$

The effect of motivation is controlled by a threshold θ, which depends on the agent's past motivation:

$$\theta_k^t = \frac{1}{\sum_{j=1}^{t-1}(\gamma^j)} \sum_{i=1}^{t-1} \left(\gamma^{t-i} M_k\right) \tag{2}$$

where the threshold for agent k at time t is a weighted sum of past motivation values, $\gamma \in [0, 1]$ being the forgetting factor, which gives more weight to recent motivations. When an agent's motivation exceeds the threshold θ, it chooses a more aggressive action, by venturing farther away from its present location, in a random direction. If the motivation is below the threshold, the exploration range is shorter.

After carrying out the chosen action, the agent updates H, which consists of two components: ${}_fH$, based on finding food during exploration, and ${}_sH$, based on social comparison:

$$H_k = (1 - s_k) \cdot {}_fH_k + s_k \cdot {}_sH_k \tag{3}$$

where s_k, agent k's sociality/food weighting parameter, is in the range of $[0, 1]$. The food-based component is computed as follows:

$$_fH_k^t = {}_fH_k^{t-1} + \alpha_k F_k^t - \beta_f \tag{4}$$

where F is the number of food units that the action yielded and α_k controls the contribution of food to agent k's hedonic well-being; β_f is the food equivalent of the agents' energy consumption per action cycle. For each agent, there is an upper bound on the maximum amount of food an agent can gain per cycle.

The social component of hedonic well-being $_sH$ for agent k at time t is computed as shown below:

$$_sH_k^t = H_k^t - \frac{1}{n_k}\sum_{j=1}^{n_k}\left(H_j^t\right) + \beta_s \tag{5}$$

where the trait n_k represents the size of agent k's social comparison group. Intuitively, an agent is happier when it is doing better than group average and less happy otherwise. β_s is the base hedonic well-being gain from socialization.

The agent's eudaimonic well-being E is then computed from its present value of H, the memory of the past values of H extending over a number of cycles, and the rates of rise and fall of H. Specifically,

$$\begin{gathered} E_k^t = \frac{1}{\Delta t_k}\sum_{i=t-\Delta t_k}^{t}\left(H_k^i + s_p\left(\frac{dH_k^i}{di}\right) + s_n\left(\frac{dH_k^i}{di}\right)\right) \\ s_p(x) = \begin{cases} {}_p\lambda_k \cdot x & x \geq 0 \\ 0 & x < 0 \end{cases} \qquad s_n(x) = \begin{cases} 0 & x \geq 0 \\ {}_n\lambda_k \cdot x & x < 0 \end{cases} \end{gathered} \tag{6}$$

where Δt_k is the extent of the memory window for agent k. The step function s_p selects the weight assigned to upswings of H and and s_n — to downswings; $_p\lambda_k$ and $_n\lambda_k$ are the respective weights. Thus, every individual can in principle value positive and negative events differently.

In parallel with computing H and E (which are subsequently fed back and used to determine motivation, as per eq. 1), the agent's fitness is updated, as follows:

$$\mathcal{F}_k^t = \sum_{i=1}^{t}(F_k^i - A_k^i - \beta_0) \tag{7}$$

Thus, the fitness $\mathcal{F}$ of agent k at time t is the total amount of food F_k^t that agent k consumed, less the cost A_k^t of its actions (with aggressive and conservative actions weighted appropriately) and a base metabolic expenditure β_0.

Experiments

Experiment setup

In each of the experiments described below, the parameter (trait) of interest was discretized as appropriate, so that equal proportions of the population carried each level

of the trait; the other parameters were kept fixed. Each experiment was repeated 10 times; each of the figures in the main text shows the means and the 95% confidence intervals for the number of carriers in the population of each level of the trait of interest, plotted against generation number.

The experiment was conducted on a quad-core computer using Matlab. The code is available at `https://www.openabm.org/model/4934`. To speed up the process, the implementation utilized all cores to achieve parallel computing.

Experiment parameters

The list of all the parameters in our model appears below.

- c in the range of $[0, 1]$ controls the contribution of hedonic and eudaimonic components to the agent's well-being. In Experiment 1, it is discretized into 11 groups in the range of $[0, 1]$. Pairwise comparison is also performed for $c = 0.3$ and $c = 0.7$.

- α_k represents how much hedonic value agent k derives from one unit of food. In Experiment 2, α is discretized to $[0.5, 1, 1.5, 2, 2.5, 3]$ and pairwise comparison is performed for $\alpha = 0.5$ and $\alpha = 2$.

- s_k represents agent k's balancing returns from food and from social comparison. The range of s_k is in $[0, 1]$ and it is discretized to $[0, 0.2, 0.4, 0.6, 0.8, 1]$ in Experiment 3. The pairwise comparison is performed for $s_k = 0.2$ and $s_k = 0.8$.

- n_k represents the size of agent k's social circle; its value in Experiment 3 is set to 8.

- Δt_k is the extent of the memory window for agent k. In Experiment 4, Δt is discretized to $[1, 2, 4, 8, 116]$ and the pairwise comparison is between values of 2 and 8.

- ${}_p\lambda_k$ is the weight of hedonic increase for agent k in the computation of eudaimonic happiness E.

- ${}_n\lambda_k$ is the weight of hedonic decrease for agent k. In Experiments 5 and 6, the pairwise comparison is conducted for ${}_p\lambda = 2,_n \lambda = 0.5$ vs. ${}_p\lambda = 0.5,_n \lambda = 2$.

- p is the proportion of poison items in the environment, in the range of $[0, 1]$. In Experiment 6, $p = 0.7$.

- β_f is the food equivalent of the agents' energy consumption per action cycle. In our experiments, $\beta_f = 2$ and $\beta_f = 5$ were tested.

- β_s is the base hedonic pleasure obtained from socialization; $\beta_s = 2$ and $\beta_s = 5$ are tested.

- d_{agg} represents the maximum "aggressive action" distance that an agent is allowed to travel when its motivation exceeds θ. In our experiments, $\max d_{agg} = 10$.

- d_{con} represents the maximum "conservative action" distance that an agent is allowed to travel when its motivation is below θ. In the experiments, $\max d_{con} = 5$.

- F_k is the lifetime food consumption of agent k. Each unit of food is worth 10 energy units.

Variables	Exp 1	Exp 2	Exp 3	Exp 4	Exp 5	Exp 6
c	–	0.5	0.5	0.5	0.5	0.5
α_k	1	–	1	1	1	1
s_k	0	0	–	0.2	0.2	0
Δt_k	8	8	8	–	8	8
${}_p\lambda_k$	1	1	1	1	–	–
${}_n\lambda_k$	1	1	1	1	–	–
p	0	0	0	0	0	0.7
β_f	2	2	2	2	2	2
β_s	2	2	2	2	2	2

Table 2. The values of the key parameters used in each experiment. An entry of "–" means that the corresponding parameter is discretized to a range of values in the experiment.

- A_k is the total number of steps agent k took in its lifetime. For each step, the energy cost is 1 or 2.

In each experiment, one of the parameters varied (as described in the main text), while the others were kept constant at their default values. The default values for "background" parameters, such as $\beta_f, \beta_s, d_{agg}, d_{con}$, were chosen so as to prevent ceiling effects and mass extinctions. One of our criteria for a "reasonable" range of those parameters was based on the percentage of agents with positive fitness: when food is scarce, $[2\%, 10\%]$; when food is average, $[30\%, 50\%]$; when food is abundant, the percentage of agents with positive fitness should be greater than 70%.

The experiments reported in the main paper involved the parameter values shown in Table 2. For further details, consult the code that is available at `https://www.openabm.org/model/4934`.
